# Correlation of gestational age and age at death in sudden infant death syndrome: another pointer to the role of critical developmental period?

**DOI:** 10.1186/s12887-024-04712-3

**Published:** 2024-04-19

**Authors:** Malgorzata Habich, Piotr Zielenkiewicz, Leszek Paczek, Pawel Szczesny

**Affiliations:** 1 Department of Bioinformatics, Institute of Biochemistry and Biophysics, Polish Academy of Sciences, ul. Pawinskiego 5A, Warsaw, 02-106 Poland; 2 Department of Immunology, Transplantology, and Internal Diseases, Medical University of Warsaw, Warsaw, Poland

**Keywords:** Sudden infant death syndrome (SIDS), Triple-risk model, Critical developmental period, Gestational age

## Abstract

**Background:**

Filiano and Kinney proposed a triple-risk model for the sudden infant death syndrome (SIDS) that involves the intersection of three risks: (1) a vulnerable infant, (2) a critical developmental period in homeostatic control, and (3) an exogenous stressor(s). The primary evidence for the role of a critical developmental period in SIDS etiology is the peak of cases around the third month of life. Independently, several studies pointed to correlation between gestational age and age at death in SIDS, but used that to assess the SIDS risk for preterm infants, ignoring further ramifications.

**Methods:**

We did a detailed analysis of CDC data spanning over two decades (1983–2011). We focused not only on the correlation between two age variables (gestational and age at death), but also on the possibility of misdiagnosis. Also, we attempted to account for potential biases in the data induced by the ICD-9/ICD-190 transition or the “Back to Sleep” campaign.

**Results:**

The peak of deaths in the third month of life, that was the main argument for the role of the critical development period, wasn’t unique to SIDS. However, we confirmed an almost linear and negative correlation between gestational age and the week of death due to SIDS. This pattern (slope of correlation < 0 and significance of correlation *p* < 0.05) is characteristic of SIDS among all diseases analyzed in the study.

**Conclusions:**

We interpret the results as the evidence of the role of the critical development period in SIDS etiology. Possibly more attention in the future research should be put to theories that are based on homeostatic control.

**Supplementary Information:**

The online version contains supplementary material available at 10.1186/s12887-024-04712-3.

## Introduction

The incidences of sudden infant death have been known since antiquity, but throughout the ages, most of the cases were attributed to accidental smothering and overlaying. As a prevention measure, at the beginning of the 20th century, cribs started to become more and more popular, but the magnitude of the problem did not substantially decrease. This was the time when the term “cot death” was coined, and physicians and scientists started to look into the etiology and risk factors. Beginning in 1969, the deaths of children younger than 12 months without another explanation of death started being classified as caused by sudden infant death syndrome (SIDS) [1]. Analysis of risk factors led to the campaign “Back to Sleep,” which started in 1994 and promoted safety measures for infant sleep in the United States. This initiative has been attributed to a 50% reduction in mortality rates. Despite these advancements, the etiology of SIDS remains unknown, and it continues to be a diagnosis of exclusion.

The same year, in 1994, Filiano and Kinney [2] proposed the “triple risk hypothesis” which became a frequently used framework in later research on SIDS. The hypothesis states that death occurs in a fatal Venn diagram of events: (1) a vulnerable child, (2) risk factors in the environment, and (3) the child is in a critical period.

Theories about mechanistic causes of death often try to answer the question of why the child was vulnerable (for example genetic factors, latent infection, etc.) in the light of known risk factors but rarely explain the critical period. This part of the hypothesis rose from the unusual pattern of death statistics. The peak of mortality due to SIDS is in the 3rd month of life, which raises questions about the nature of the cause. If the cause is congenital, why does it activate in the 3rd month? Is there something pivotal during this period? Or are we missing something, and the critical period is only a statistical artifact?

One of the risk factors of SIDS is prematurity [3]. Prematurity can be connected with intestine development issues [4], abnormal brain development [5], respiratory problems [6], viral infection susceptibility [7], or increased risk of cardiovascular events [8]. Because of the delayed gross development of premature babies [9] pediatricians use adjusted age to track milestones. Because consequences of this event can vary and be as well short term and long term, it is easy to overlook this risk factor as an independent variable. Even if the correlation between gestational age and age at death in SIDS was observed [10], there were only a few studies that interpreted this observation beyond risk factor alone [11–13]. None of them linked this explicitly to the triple risk hypothesis or any other framework that would point to the role of development in SIDS etiology.

In this study, we analyzed CDC data on child mortality from 1986 to 2005. We showed that it is not the peak of deaths in the third month of life that distinguishes SIDS from other causes of death. It is the distribution of the adjusted age of death that is a characteristic property of this condition. As such, this might point to the underexplored role of homeostatic control in the etiology of SIDS.

## Materials and methods

### Data sources

The data for the study was obtained from the CDC database (https://www.cdc.gov/nchs/data_access/vitalstatsonline.htm) Birth Cohort Linked Birth – Infant Death Data Files. At the time of analysis, data were provided for years from 1983 to 2011 with a gap between 1992 and 1994. The data was deciphered based on the User’s Guide manual.

### Data harmonization

The contents of the source database was changing yearly, so the fields of interest were translated into a common format, for example days into weeks etc. The biggest problem was the change of the “cause of death” field, which used ICD codes in version 9 before 1999 and version 10 from then on. Although there is an official conversion guide between the two formats, the correspondence is ambiguous, being a many-to-many relationship, which is difficult to distinguish without specialist knowledge. To automate the process of unification of data, we created new codes of causes of death which were joining analogous codes from ICD-9 and ICD-10.

We extracted unique ICD9 and ICD10 codes from CDC data and treated them as nodes in a graph with paths if the codes were listed as equivalence based on Diagnosis Code Set General Equivalence Mappings ICD-10-CM to ICD-9-CM and ICD-9-CM to ICD-10-CM Documentation and User’s Guide (https://ftp.cdc.gov/pub/health_statistics/nchs/publications/ICD10CM/2018/Dxgem_guide_2018.pdf). From this graph, we extracted connected components and named them our new joined codes. Most of the created codes consisted of a few ICD-9 and ICD-10 codes, which could be easily translated into more general categories like bacterial meningitis instead of listing the specific type of bacteria.

As a result of the data harmonization procedure, in this study, we operate not on distinct diseases but rather on groups of very similar (in terms of etiology) diseases. The list of groups and associated codes is in the supplementary material.

### Data analysis

To minimize the influence of trends in SIDS diagnosis and changes in autopsy protocols on our analysis, we limited data to the years around the “Back to Sleep” campaign and divided these years into three categories: before (1986–1991), during (1995–1999) and after (2000–2005). During the analysis of death patterns of other causes than SIDS, we limited the analysis to causes that had more than 20 cases per year to exclude ultra-rare diseases which are difficult to describe statistically. All analysis and visualizations were made using Python 3.0 packages including SciPy, pandas, and seaborn.

## Results

### SIDS or not SIDS, that is the question

**Fig. 1:**
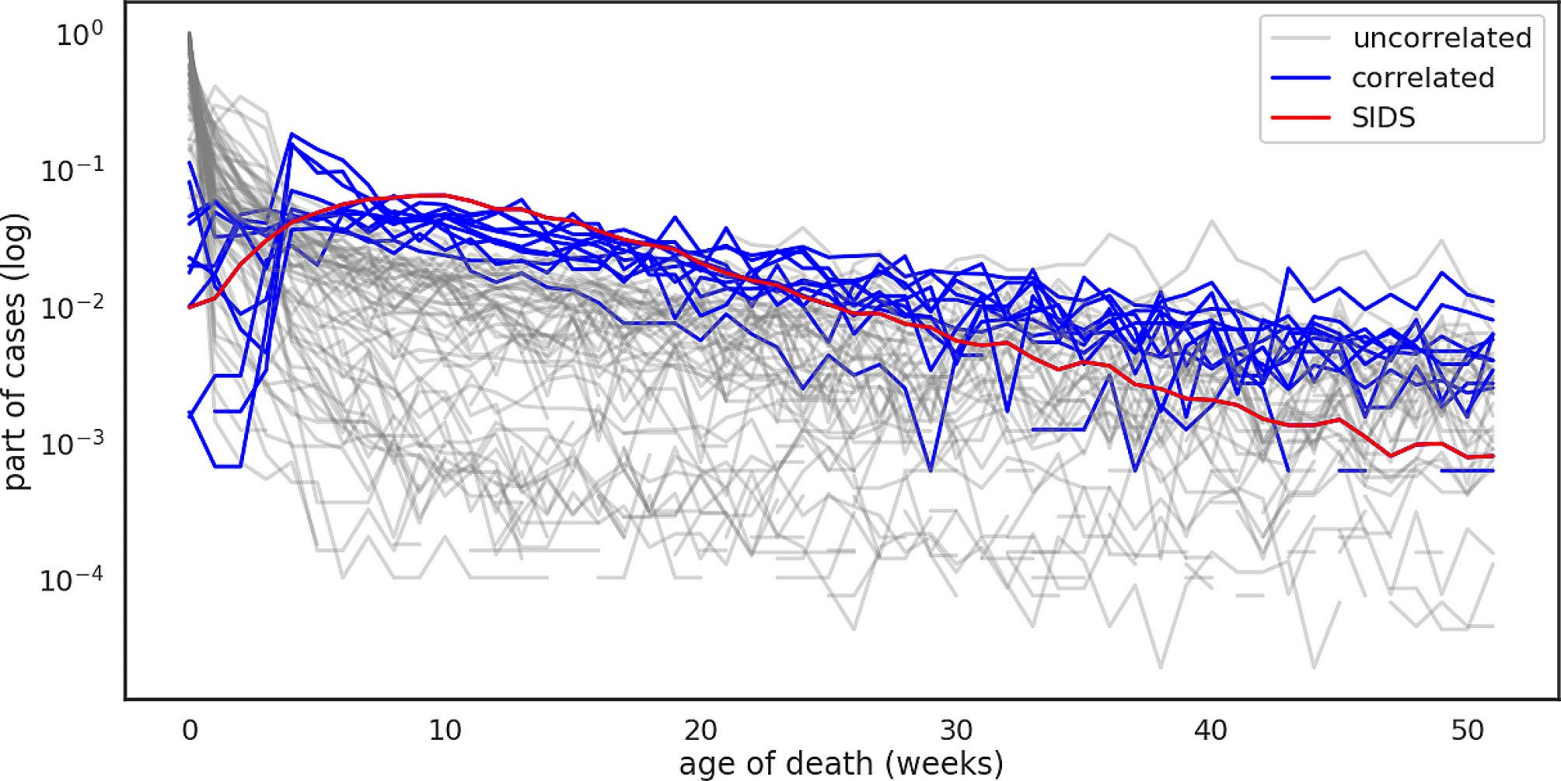
Normalized (sum of cases of every week divided by the sum of all cases of the cause) deaths per week of all analyzed groups of conditions. The sum of cases of every week of death was divided by the sum of all cases of the cause. The red line indicates SIDS, and the blue lines represent 13 causes that correlated with SIDS (Pearson correlation coefficient > 0.5). The gray lines represent uncorrelated causes and other causes of death

As the basis for the critical development period was the observation of the peak of SIDS cases in the 3rd month of life, we sought out to understand this property, especially in the light of newer publications questioning the existence of SIDS as a separate condition. The simple correlation analysis identified 12 cause groups that had at least a 0.5 correlation (Pearson standard correlation coefficient test) with SIDS (Fig. 1), from all 77 disease groups that were available after our harmonization procedure described in the Methods section. The list of 12 disease groups that have similar death patterns is: Ill-defined and unknown cause of mortality, Cardiac arrest - cause unspecified, Acute vascular disorders of intestine, Chronic respiratory disease originating in the perinatal period, Acute bronchiolitis, Viral pneumonia, Hepatic failure, Sepsis - unspecified organism, Group of intestinal disorders, Anoxic brain damage - not elsewhere classified, Sepsis due to Gram-negative organisms and of course SIDS.

**Fig. 2:**
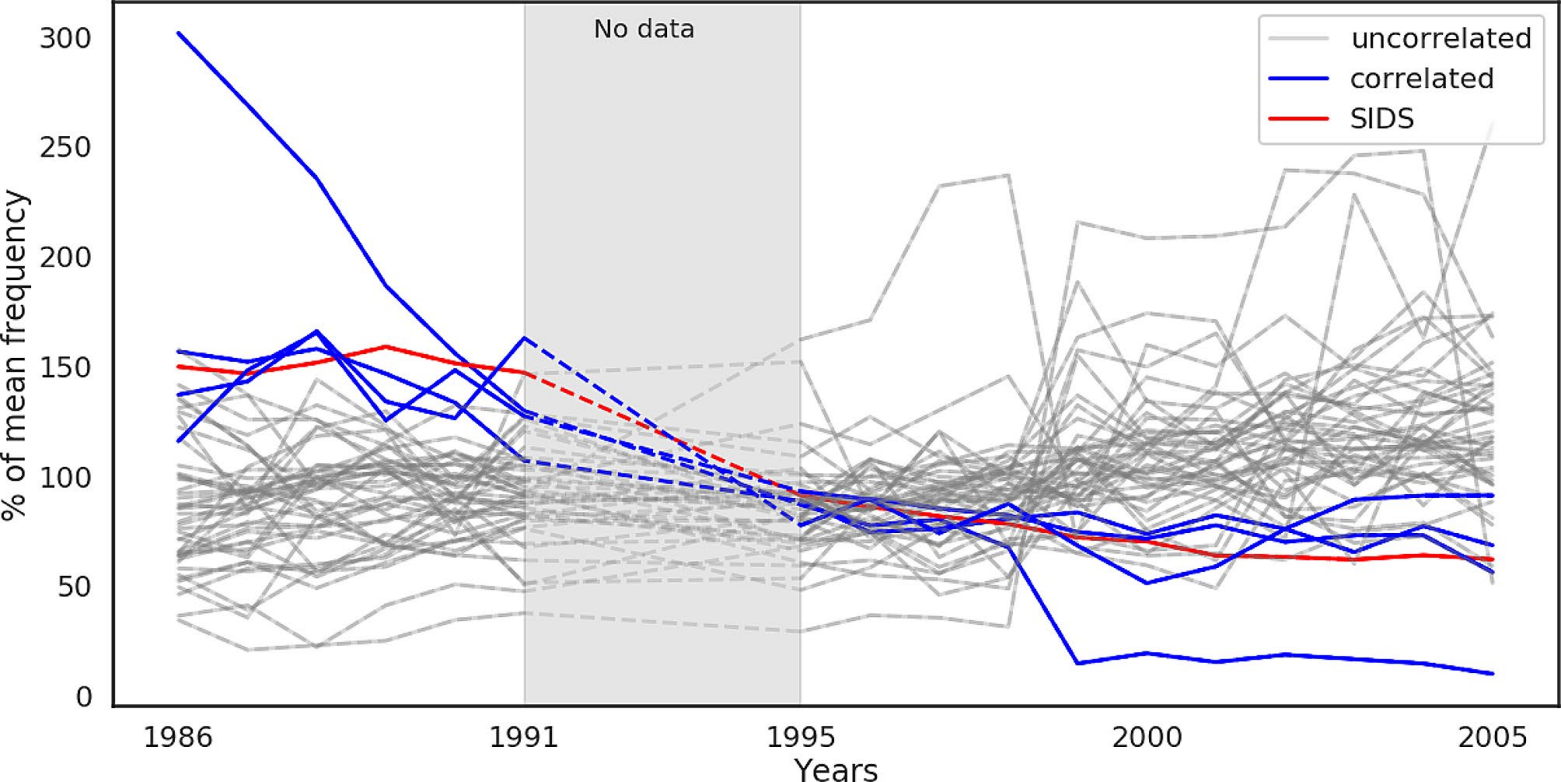
Normalized annual frequency of deaths causes (expressed as a percentage of the average level of those causes through time) between 1986 and 2005. Grey area shows missing CDC data (years 1992–1994). Red line is SIDS and blue lines are 8 causes that correlated with SIDS in this time course and peaked in the 3rd month (blue lines on the Fig. 1). Grey lines are all other causes

Another statistical feature of SIDS is the drop of mortality due to the “Back to Sleep” campaign. Here we test for a common argument of misdiagnosis. However, since a drop in mortality over time is expected because of the advances in medicine and childcare, we suspected that most of the diseases would meet the criteria. From 76 disease groups that we analyzed, 33 met the criteria of correlated drop in mortality (positive correlation with SIDS / p-value < 0.005 / Pearson standard correlation coefficient test). We compared these diseases with the 13 disease groups which we have already found to peak in 3rd month, and we ended up with 4 groups which are candidates for further investigation (Fig. 2): Cardiac arrest - cause unspecified, Chronic respiratory disease originating in the perinatal period, Viral pneumonia, Anoxic brain damage - not elsewhere classified.

### Gestation age negatively correlates with age of death for SIDS

**Fig. 3:**
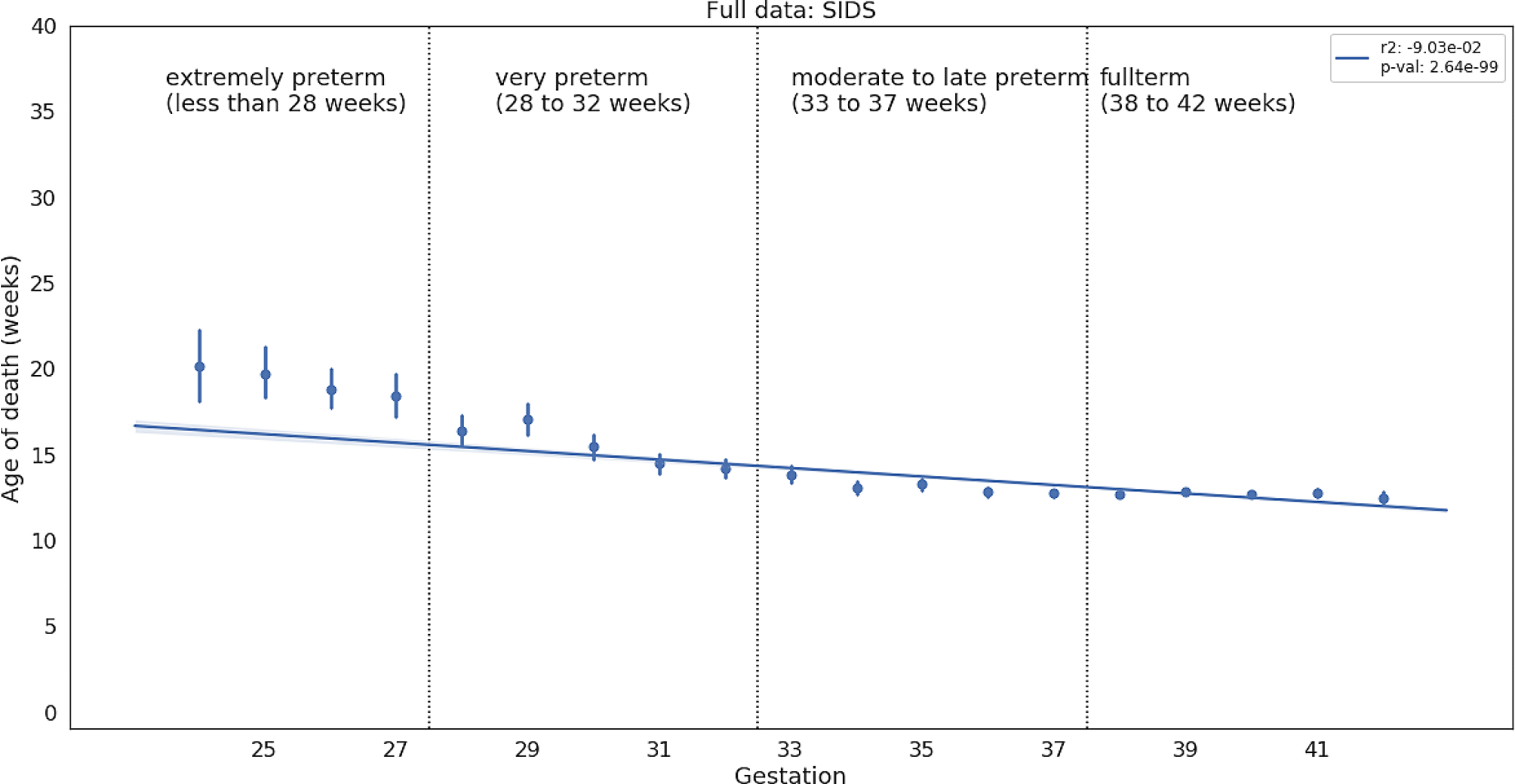
Mean age of death and standard deviation for every week of gestation for SIDS. Blue line is a regression line with a 95% confidence interval shaded around

We compared gestation age with age of death for all disease groups. We found only four causes of death where preterms die later than full term newborns (with the following conditions: linear regression with slope less than 0 and p-value less than 0.05): SIDS, Necrotizing enterocolitis in newborn, Fetal and newborn aspiration, and Acute bronchiolitis. Out of those additional three, acute bronchiolitis is the only disease that has a peak of deaths during the third month, as noted in the previous section. The other two diseases usually occur before the newborn is discharged from the hospital and goes home. The relationship between gestational age and age of death for all for SIDS is shown in Fig. 3. and for three other diseases is available in supplementary material as Supplementary Fig. 1.

**Fig. 4:**
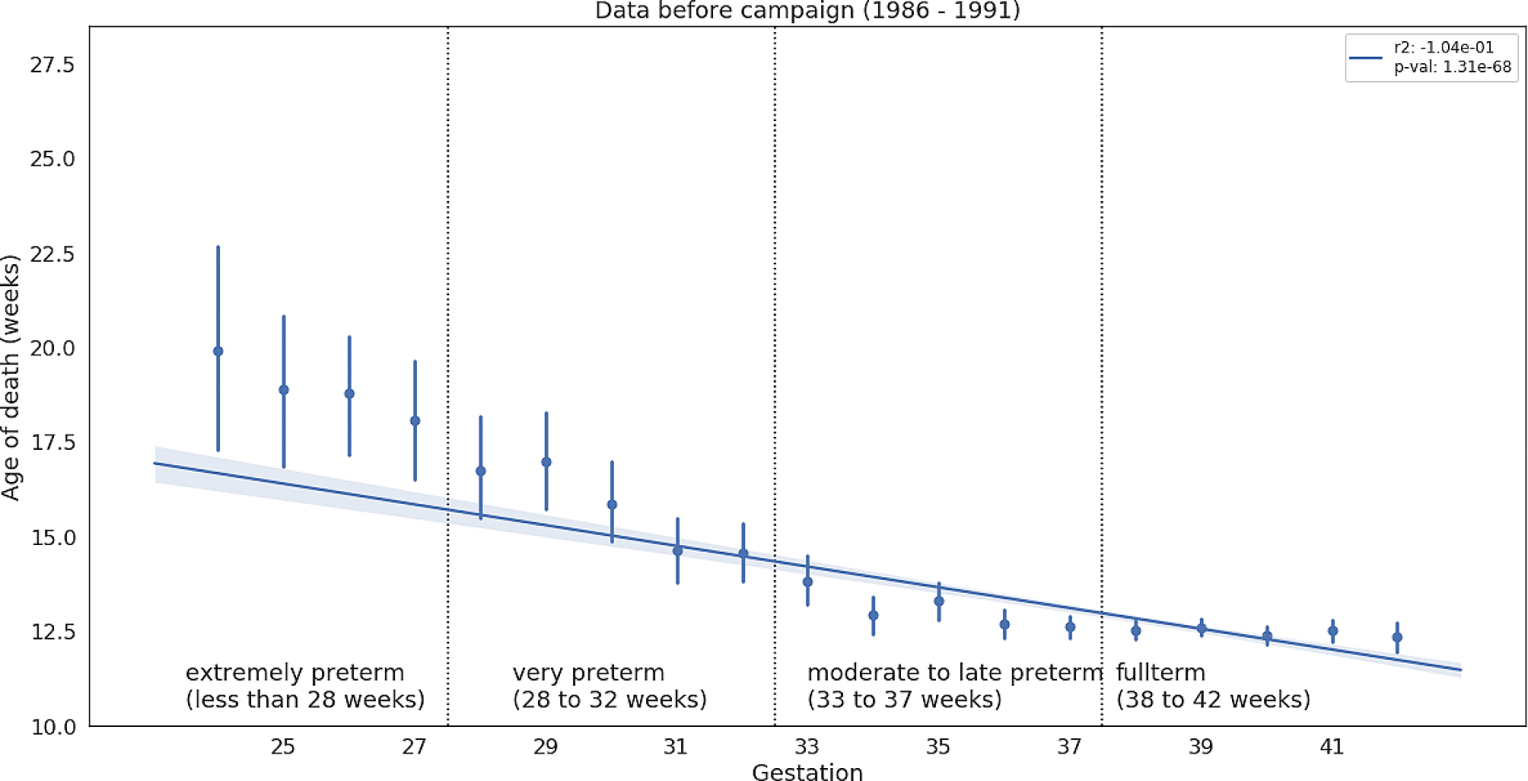
Mean age of death and standard deviation for every week of gestation for before the campaign “Back to sleep”. Blue line is a regression line with a 95% confidence interval shaded around. P-value is in the legend box

To verify whether preterm infants die later than full-term newborns in the case of SIDS, we repeated the analysis by splitting the data into groups: before, during, and after the “Back to Sleep” campaign. This was done to remove the possibility that changes in diagnostics were influencing the results. The relationship remains significant in all three groups separately - as shown in Fig. 4 (before the campaign) and in Supplementary Material Fig. 2 (during and after the campaign).

Additionally, to remove the effect of transitioning between two ICD versions (and merging, replacing or removing some conditions), we repeated the analysis for ICD-9 and ICD-10 separately. In addition to SIDS, which consistently comes up in all subsets as statistically significant, we found Necrotizing enterocolitis in newborn, Fetal and newborn aspiration, and Poisoning in ICD-9, and Other bacterial sepsis of newborn, Necrotizing enterocolitis in newborn, Car accident, Suffocation and Assault by unspecified means in ICD-10.

## Discussion

In this study, we attempted to provide evidence for the role of the critical development period in SIDS. Several other diseases also have the peak of deaths in the 3rd month when taken into account unadjusted age. Out of 12 disease groups, four were infection-related: Acute bronchiolitis, Viral pneumonia, Sepsis - unspecified organism, Sepsis due to Gram-negative organisms. Finding infections in this group doesn’t seem to be an accident. Goldwater did a detailed comparison of clinicopathological and epidemiological features between SIDS and deaths from sepsis, and he found a staggering number of similarities [14]. Infections could act as a common SIDS trigger, and the overlap seen in unadjusted age of death might represent common infection time.

However, we found that the inverse and strong relationship between age of death and gestational age is quite characteristic for SIDS. The relationship holds true irrespectively of the way we subset the data. Neither updates to SIDS diagnostics nor the awareness of the condition explain the relationship we observed. Our results are more robust than the ones provided by Malloy in 2013 [3], because we went into weekly data granularity. From the infection-related group found earlier, only acute bronchiolitis shares this feature and that specific group of diseases together with SIDS might be considered a subject for further studies, for instance with respect to differential diagnosis.

We ran statistical tests over the full span of values of age of death to avoid spurious correlations. However, as can be seen on Figs. 3 and 4, the correlation starts to weaken when we enter the late preterm range. We believe this further strengthens the evidence for the role of the critical development period in homeostatic control or gross development, especially if we take into account observations of delayed development of motor skills after the “Back to Sleep” campaign [15] or association of gross development rate with birth month [16].

While a lot of evidence points to autopsy results overlapping between SIDS and infections [17], as discussed above, we didn’t see such an overlap when analyzing the mortality data in multiple ways. For instance, SIDS is diagnosed by excluding other conditions and is sensitive to changes in diagnostic methods. Similar mortality patterns may suggest that SIDS is actually a group of undiagnosed cases of one of the diseases on the list. If that were true, then the decrease in SIDS mortality due to the back-to-sleep campaign would also be seen in the statistics for that disease. However, as seen in our results, various approaches to subsetting the data didn’t yield the same results.

Also, if SIDS was in fact some other disease, we would expect that the role of the critical development period was discovered independently for this other condition. We found quite a few disease groups that have a similar pattern to SIDS in various subsets of the data. From these groups, the group of intestine diseases is linked to infant development. The possible mechanism would involve gut microbiota and similar suggestions were made already in case of SIDS [18, 19] as well in case of other diseases [20]. Also, there are some reports pointing out the influence of birth age on the course of bronchiolitis [21]. However, we didn’t find any evidence in these studies that would directly point to delayed development of premature infants.

As some authors point out [17, 22], there seems to be a blind spot in the research on the critical developmental period in homeostatic control. There’s some evidence for the role of brain stem development [23] and this avenue was explored for years. However, as Guntheroth and Spiers point out, brain stem development theory would imply more deaths at the neonatal stage than at the age of 1–6 months (SIDS peak), as the neonatal stage is more “unstable” physiologically [24]. Other hypotheses related to cardiac control [25], chronic hypoxia [26], or critical diaphragm failure [27] seem to be underexplored. Given how tightly these hypotheses connect to infection as a trigger, it seems that homeostatic control of the immune system should be a promising avenue for further SIDS research. And indeed, recent profiling of lungs of SIDS cases suggests that an impaired maturation of the immune system resulting in insufficient response to respiratory pathogens might be the SIDS trigger [28]. Among cytokines that were significantly decreased in Qu’s study was IL-1RA, the interleukin-1 receptor antagonist, which was shown to protect against white matter inflammation in near-term fetal sheep exposed to lipopolysaccharide [29]. Therefore, one of the plausible hypotheses could be that SIDS is the result of the race between inflammation-mediated respiratory depression due to homeostatic plasticity that is more frequently observed in premature infants [30] and rapid development of diaphragm in SIDS [27]. Also, impaired immune system homeostasis could potentially explain changes in gut microbiota mentioned earlier. However, the exact mechanism remains to be elucidated because the temporal profile of immune system development isn’t well understood.

We also considered alternative explanations for the observed correlation. However, we couldn’t find supporting data for behavioral differences that would explain it (such as gradually delayed exposure of a preterm infant to the outside environment influencing putative infection rate). Also, one could argue that delayed motor development would potentially decrease a chance an infant can turn into a prone position during sleep. However, that doesn’t fully explain SIDS victims that died in supine position.

We hope that our study will inspire researchers to reconsider infant development trajectory in the attempts to decode the mechanism of SIDS.

### Electronic supplementary material

Below is the link to the electronic supplementary material.

Supplementary Material 1

## Data Availability

The code used during the current study is available in the GitHub repository: https://github.com/habich/SIDS_critical_period.
