# Supplementary material for: Correlation of gestational age and age at death in sudden infant death syndrome: another pointer to the role of critical developmental period?
Source: BMC Pediatr. 2024 Apr 19;24:259. doi: 10.1186/s12887-024-04712-3 (PMC11027530; doi:10.1186/s12887-024-04712-3)
Supplement: Supplementary file 1 — Supplementary Material 1 [file 12887_2024_4712_MOESM1_ESM.docx]

Supplementary Material

**Correlation of gestational age and age at death in Sudden Infant Death Syndrome: another pointer to the role of critical developmental period?**

Malgorzata Habich 1, Piotr Zielenkiewicz 1, Leszek Paczek 1,2, Pawel Szczesny 1*

*** Correspondence:** Pawel Szczesny [szczesny.pawel@gmail.com](mailto:szczesny.pawel@gmail.com)

A:


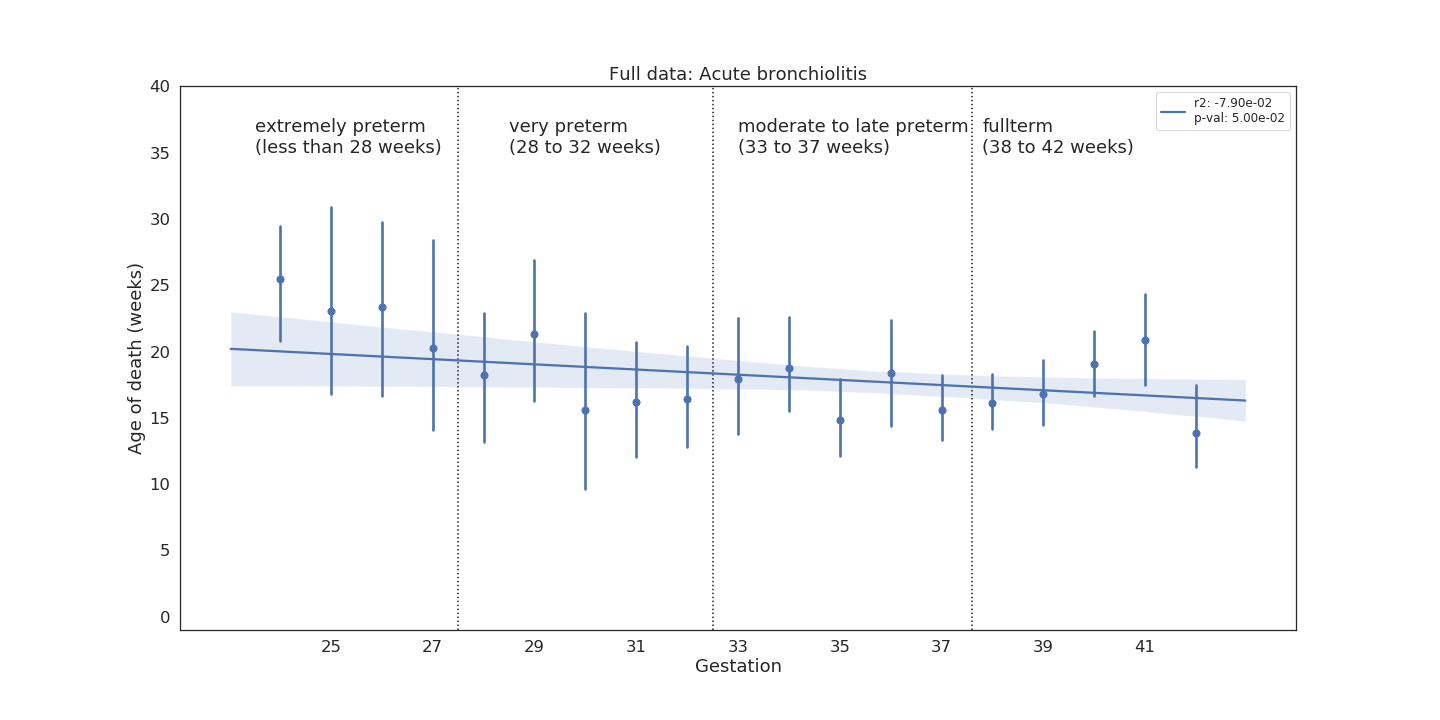


B:


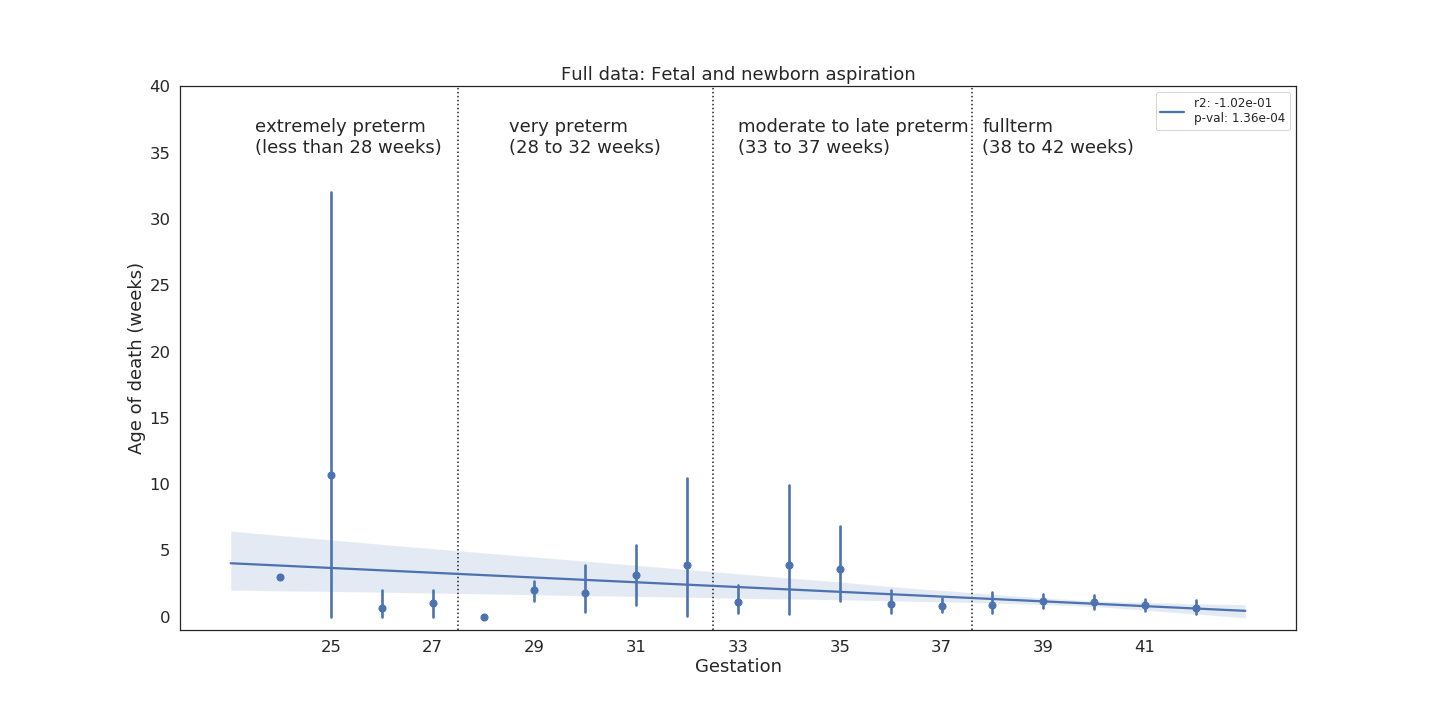
C:


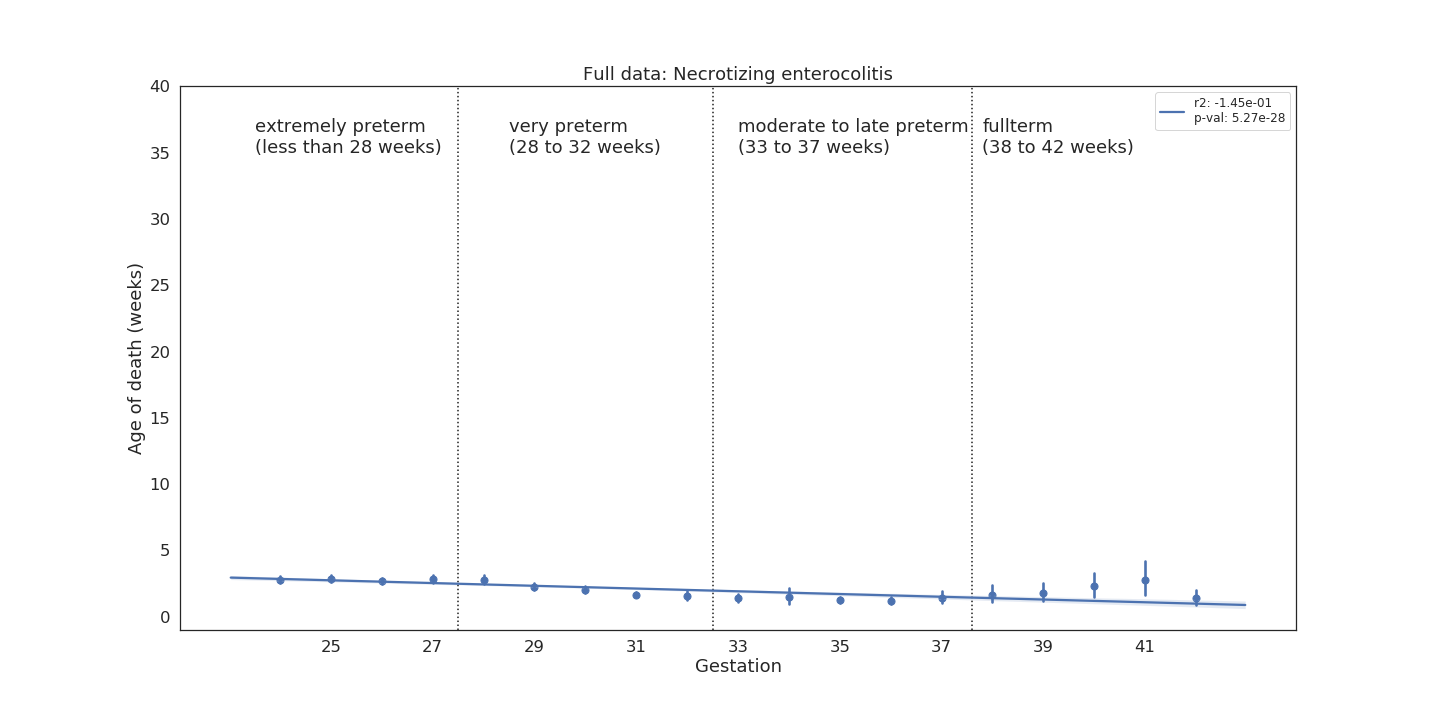


Fig 1: Mean age of death and standard deviation for every week of gestation for A: Acute bronchiolitis, B: Fetal and newborn aspiration and C: Necrotizing enterocolitis. Blue line is a regression line with a 95% confidence interval shaded around . Y-axis was kept constant between panels for easy comparison.

A:


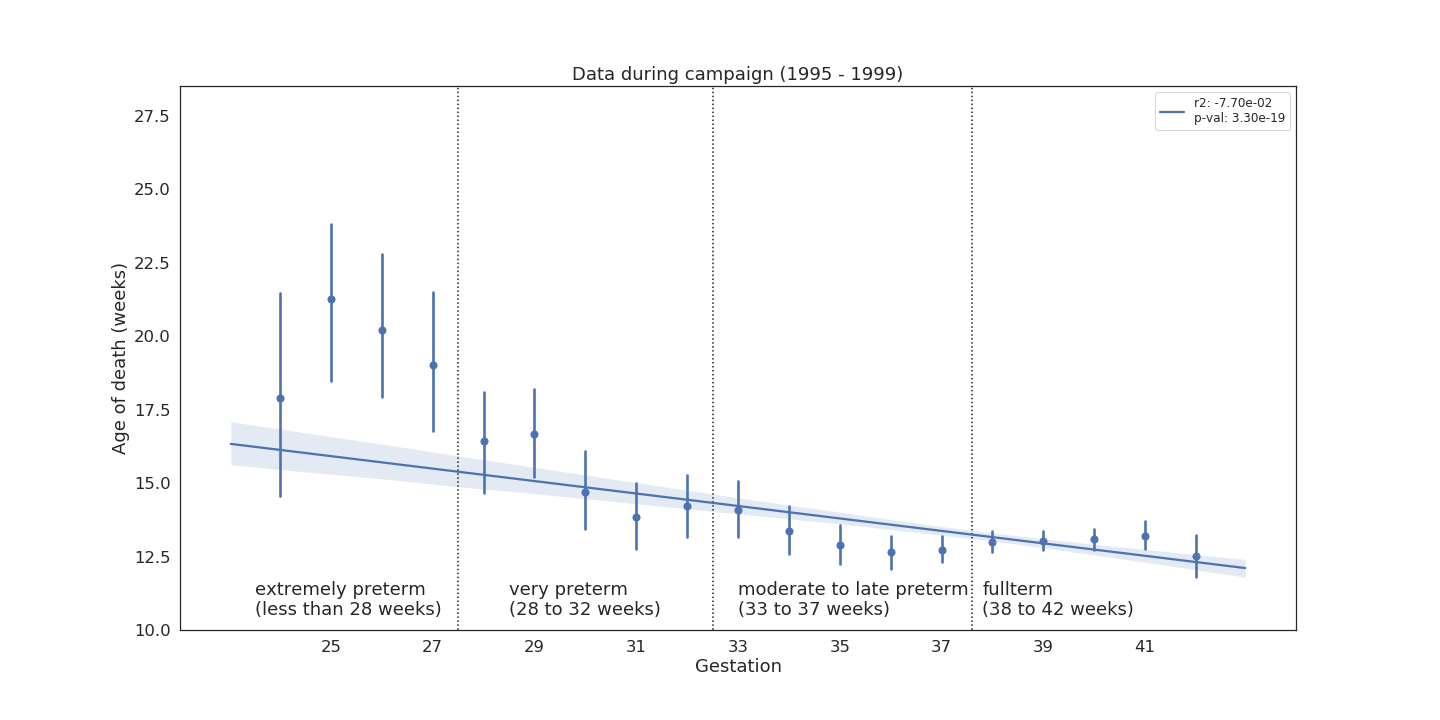
B:


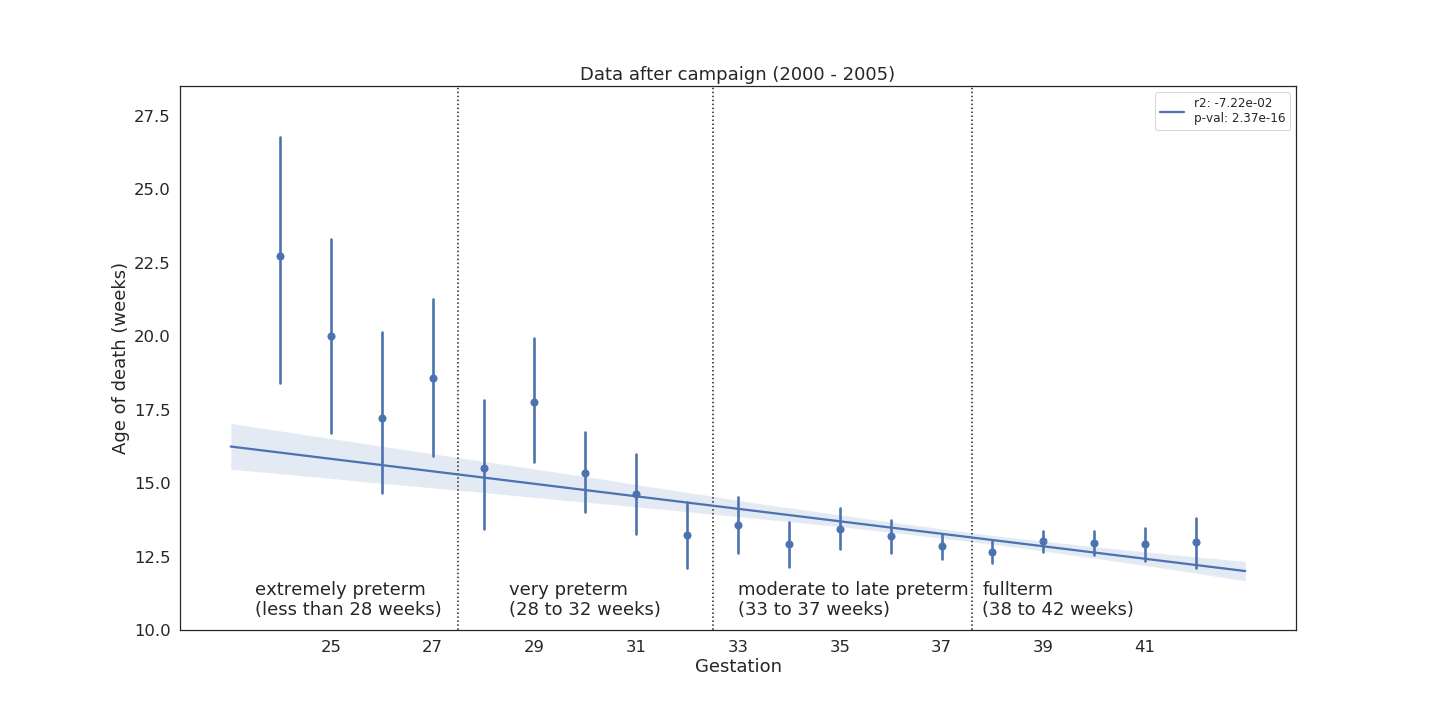


Fig 2 Mean age of death and standard deviation for every week of gestation for SIDS divided into periods during (A) and after (B ) campaign back to sleep. Blue line is a regression line with a 95% confidence interval shaded around. P-value is in the legend box.

# Supplementary Table: List of disease groups with their ICD-10 codes.

| 1 | P280 | Primary atelectasis of newborn |
| --- | --- | --- |
| 2 | P070 | Extremely low birth weight newborn |
| 2 | P071 | Other low birth weight newborn |
| 2 | P073 | Preterm [premature] newborn [other] |
| 3 | R060 | Dyspnea |
| 3 | J80 | Acute respiratory distress syndrome |
| 3 | P284 | Other apnea of newborn |
| 3 | J961 | Chronic respiratory failure |
| 3 | J984 | Other disorders of lung |
| 3 | P288 | Other specified respiratory conditions of newborn |
| 3 | P228 | Other respiratory distress of newborn |
| 3 | P229 | Respiratory distress of newborn, unspecified |
| 3 | P243 | Neonatal aspiration of milk and regurgitated food |
| 3 | R064 | Hyperventilation |
| 3 | J969 | Respiratory failure, unspecified |
| 3 | J960 | Acute respiratory failure |
| 3 | P285 | Respiratory failure of newborn |
| 3 | R068 | Other abnormalities of breathing |
| 3 | P283 | Primary sleep apnea of newborn |
| 4 | P015 | Newborn affected by multiple pregnancy |
| 5 | P968 | Other specified conditions originating in the perinatal period |
| 5 | P290 | Neonatal cardiac failure |
| 5 | P960 | Congenital renal failure |
| 5 | P298 | Other cardiovascular disorders originating in the perinatal period |
| 5 | P294 | Transient myocardial ischemia in newborn |
| 5 | P291 | Neonatal cardiac dysrhythmia |
| 5 | P008 | Newborn affected by other maternal conditions |
| 5 | P292 | Neonatal hypertension |
| 5 | P299 | Cardiovascular disorder originating in the perinatal period, unspecified |
| 5 | P948 | Other disorders of muscle tone of newborn |
| 5 | P942 | Congenital hypotonia |
| 6 | P011 | Newborn affected by premature rupture of membranes |
| 8 | P018 | Newborn affected by other maternal complications of pregnancy |
| 9 | P109 | Unspecified intracranial laceration and hemorrhage due to birth injury |
| 9 | P529 | Intracranial (nontraumatic) hemorrhage of newborn, unspecified |
| 9 | P526 | Cerebellar (nontraumatic) and posterior fossa hemorrhage of newborn |
| 9 | P119 | Birth injury to central nervous system, unspecified |
| 9 | P108 | Other intracranial lacerations and hemorrhages due to birth injury |
| 9 | P110 | Cerebral edema due to birth injury |
| 9 | P100 | Subdural hemorrhage due to birth injury |
| 9 | P524 | Intracerebral (nontraumatic) hemorrhage of newborn |
| 9 | P528 | Other intracranial (nontraumatic) hemorrhages of newborn |
| 9 | P112 | Unspecified brain damage due to birth injury |
| 9 | P101 | Cerebral hemorrhage due to birth injury |
| 10 | P027 | Newborn affected by chorioamnionitis |
| 11 | P361 | Sepsis of newborn due to other and unspecified streptococci |
| 11 | P363 | Sepsis of newborn due to other and unspecified staphylococci |
| 11 | P394 | Neonatal skin infection |
| 11 | P393 | Neonatal urinary tract infection |
| 11 | P399 | Infection specific to the perinatal period, unspecified |
| 11 | P362 | Sepsis of newborn due to Staphylococcus aureus |
| 11 | P364 | Sepsis of newborn due to Escherichia coli |
| 11 | P368 | Other bacterial sepsis of newborn |
| 11 | P369 | Bacterial sepsis of newborn, unspecified |
| 11 | P398 | Other specified infections specific to the perinatal period |
| 11 | P360 | Sepsis of newborn due to streptococcus, group B |
| 11 | P392 | Intra-amniotic infection affecting newborn, not elsewhere classified |
| 12 | R99 | Ill-defined and unknown cause of mortality |
| 13 | P523 | Unspecified intraventricular (nontraumatic) hemorrhage of newborn |
| 13 | P102 | Intraventricular hemorrhage due to birth injury |
| 13 | P520 | Intraventricular (nontraumatic) hemorrhage, grade 1, of newborn |
| 13 | P522 | Intraventricular (nontraumatic) hemorrhage, grade 3 and grade 4, of newborn |
| 13 | P521 | Intraventricular (nontraumatic) hemorrhage, grade 2, of newborn |
| 15 | P021 | Newborn affected by other forms of placental separation and hemorrhage |
| 16 | P012 | Newborn affected by oligohydramnios |
| 17 | I469 | Cardiac arrest, cause unspecified |
| 19 | P252 | Pneumomediastinum originating in the perinatal period |
| 19 | P251 | Pneumothorax originating in the perinatal period |
| 19 | P250 | Interstitial emphysema originating in the perinatal period |
| 19 | P253 | Pneumopericardium originating in the perinatal period |
| 19 | P258 | Other conditions related to interstitial emphysema originating in the perinatal period |
| 20 | Q030 | Malformations of aqueduct of Sylvius |
| 20 | Q039 | Congenital hydrocephalus, unspecified |
| 20 | Q038 | Other congenital hydrocephalus |
| 20 | Q031 | Atresia of foramina of Magendie and Luschka |
| 22 | P375 | Neonatal candidiasis |
| 24 | P268 | Other pulmonary hemorrhages originating in the perinatal period |
| 24 | P269 | Unspecified pulmonary hemorrhage originating in the perinatal period |
| 24 | P261 | Massive pulmonary hemorrhage originating in the perinatal period |
| 25 | P271 | Bronchopulmonary dysplasia originating in the perinatal period |
| 25 | P270 | Wilson-Mikity syndrome |
| 25 | P279 | Unspecified chronic respiratory disease originating in the perinatal period |
| 25 | P278 | Other chronic respiratory diseases originating in the perinatal period |
| 27 | K550 | Acute vascular disorders of intestine |
| 744 | P77 | Necrotizing enterocolitis of newborn |
| 29 | Q000 | Anencephaly |
| 32 | Q333 | Agenesis of lung |
| 32 | Q332 | Sequestration of lung |
| 32 | Q336 | Congenital hypoplasia and dysplasia of lung |
| 34 | Q913 | Trisomy 18, unspecified |
| 34 | Q912 | Trisomy 18, translocation |
| 34 | Q911 | Trisomy 18, mosaicism (mitotic nondisjunction) |
| 35 | Q897 | Multiple congenital malformations, not elsewhere classified |
| 36 | N179 | Acute kidney failure, unspecified |
| 38 | Q019 | Encephalocele, unspecified |
| 38 | Q012 | Occipital encephalocele |
| 38 | Q018 | Encephalocele of other sites |
| 39 | R95 | SIDS |
| 40 | Q249 | Congenital malformation of heart, unspecified |
| 41 | J210 | Acute bronchiolitis due to respiratory syncytial virus |
| 41 | J218 | Acute bronchiolitis due to other specified organisms |
| 41 | J219 | Acute bronchiolitis, unspecified |
| 42 | Q602 | Renal agenesis, unspecified |
| 42 | Q601 | Renal agenesis, bilateral |
| 42 | Q605 | Renal hypoplasia, unspecified |
| 42 | Q606 | Potter's syndrome |
| 42 | Q604 | Renal hypoplasia, bilateral |
| 42 | Q600 | Renal agenesis, unilateral |
| 43 | Q917 | Trisomy 13, unspecified |
| 43 | Q915 | Trisomy 13, mosaicism (mitotic nondisjunction) |
| 43 | Q914 | Trisomy 13, nonmosaicism (meiotic nondisjunction) |
| 43 | Q916 | Trisomy 13, translocation |
| 45 | P832 | Hydrops fetalis not due to hemolytic disease |
| 46 | Q213 | Tetralogy of Fallot |
| 50 | J100 | Influenza due to other identified influenza virus with pneumonia |
| 50 | J129 | Viral pneumonia, unspecified |
| 50 | J110 | Influenza due to unidentified influenza virus with pneumonia |
| 51 | Q999 | Chromosomal abnormality, unspecified |
| 58 | Q927 | Triploidy and polyploidy |
| 58 | Q925 | Duplications with other complex rearrangements |
| 58 | Q928 | Other specified trisomies and partial trisomies of autosomes |
| 58 | Q921 | Whole chromosome trisomy, mosaicism (mitotic nondisjunction) |
| 58 | Q929 | Trisomy and partial trisomy of autosomes, unspecified |
| 58 | Q932 | Chromosome replaced with ring, dicentric or isochromosome |
| 61 | Q234 | Hypoplastic left heart syndrome |
| 62 | Q252 | Atresia of aorta |
| 62 | Q253 | Supravalvular aortic stenosis |
| 62 | Q251 | Coarctation of aorta |
| 62 | Q258 | Other congenital malformations of other great arteries |
| 62 | Q254 | Other congenital malformations of aorta |
| 63 | Q790 | Congenital diaphragmatic hernia |
| 63 | Q791 | Other congenital malformations of diaphragm |
| 64 | Q872 | Congenital malformation syndromes predominantly involving limbs |
| 64 | Q878 | Other specified congenital malformation syndromes, not elsewhere classified |
| 64 | Q873 | Congenital malformation syndromes involving early overgrowth |
| 64 | Q874 | Marfan's syndrome |
| 64 | Q871 | Congenital malformation syndromes predominantly associated with short stature |
| 64 | Q898 | Other specified congenital malformations |
| 74 | Q212 | Atrioventricular septal defect |
| 78 | Q257 | Other congenital malformations of pulmonary artery |
| 78 | Q255 | Atresia of pulmonary artery |
| 78 | Q256 | Stenosis of pulmonary artery |
| 79 | Q214 | Aortopulmonary septal defect |
| 79 | Q206 | Isomerism of atrial appendages |
| 79 | Q218 | Other congenital malformations of cardiac septa |
| 79 | Q203 | Discordant ventriculoarterial connection |
| 79 | Q208 | Other congenital malformations of cardiac chambers and connections |
| 79 | Q201 | Double outlet right ventricle |
| 79 | Q202 | Double outlet left ventricle |
| 81 | I428 | Other cardiomyopathies |
| 81 | I425 | Other restrictive cardiomyopathy |
| 81 | I420 | Dilated cardiomyopathy |
| 81 | I429 | Cardiomyopathy, unspecified |
| 84 | Q780 | Osteogenesis imperfecta |
| 84 | Q781 | Polyostotic fibrous dysplasia |
| 84 | Q776 | Chondroectodermal dysplasia |
| 84 | Q785 | Metaphyseal dysplasia |
| 84 | Q782 | Osteopetrosis |
| 84 | Q788 | Other specified osteochondrodysplasias |
| 84 | Q773 | Chondrodysplasia punctata |
| 84 | Q789 | Osteochondrodysplasia, unspecified |
| 86 | P240 | Meconium aspiration |
| 86 | P249 | Neonatal aspiration, unspecified |
| 86 | P242 | Neonatal aspiration of blood |
| 86 | P248 | Other neonatal aspiration |
| 86 | P241 | Neonatal aspiration of (clear) amniotic fluid and mucus |
| 87 | Q262 | Total anomalous pulmonary venous connection |
| 87 | Q269 | Congenital malformation of great vein, unspecified |
| 87 | Q264 | Anomalous pulmonary venous connection, unspecified |
| 87 | Q263 | Partial anomalous pulmonary venous connection |
| 87 | Q268 | Other congenital malformations of great veins |
| 88 | Q210 | Ventricular septal defect |
| 97 | K729 | Hepatic failure, unspecified |
| 97 | K721 | Chronic hepatic failure |
| 97 | K720 | Acute and subacute hepatic failure |
| 101 | Q250 | Patent ductus arteriosus |
| 104 | Q283 | Other malformations of cerebral vessels |
| 104 | Q265 | Anomalous portal venous connection |
| 104 | Q282 | Arteriovenous malformation of cerebral vessels |
| 104 | Q273 | Arteriovenous malformation (peripheral) |
| 104 | Q271 | Congenital renal artery stenosis |
| 104 | Q278 | Other specified congenital malformations of peripheral vascular system |
| 104 | P293 | Persistent fetal circulation |
| 104 | Q279 | Congenital malformation of peripheral vascular system, unspecified |
| 104 | Q288 | Other specified congenital malformations of circulatory system |
| 105 | P010 | Newborn affected by incompetent cervix |
| 108 | Q043 | Other reduction deformities of brain |
| 108 | Q040 | Congenital malformations of corpus callosum |
| 108 | Q042 | Holoprosencephaly |
| 108 | Q041 | Arhinencephaly |
| 109 | Q200 | Common arterial trunk |
| 111 | A419 | Sepsis, unspecified organism |
| 121 | Q899 | Congenital malformation, unspecified |
| 122 | Q244 | Congenital subaortic stenosis |
| 122 | Q238 | Other congenital malformations of aortic and mitral valves |
| 122 | Q240 | Dextrocardia |
| 122 | Q248 | Other specified congenital malformations of heart |
| 122 | Q246 | Congenital heart block |
| 122 | Q239 | Congenital malformation of aortic and mitral valves, unspecified |
| 122 | Q245 | Malformation of coronary vessels |
| 122 | Q242 | Cor triatriatum |
| 129 | Q751 | Craniofacial dysostosis |
| 129 | Q753 | Macrocephaly |
| 129 | Q870 | Congenital malformation syndromes predominantly affecting facial appearance |
| 129 | Q749 | Unspecified congenital malformation of limb(s) |
| 129 | Q798 | Other congenital malformations of musculoskeletal system |
| 129 | Q799 | Congenital malformation of musculoskeletal system, unspecified |
| 129 | Q688 | Other specified congenital musculoskeletal deformities |
| 129 | Q759 | Congenital malformation of skull and face bones, unspecified |
| 129 | Q750 | Craniosynostosis |
| 129 | Q740 | Other congenital malformations of upper limb(s), including shoulder girdle |
| 129 | Q758 | Other specified congenital malformations of skull and face bones |
| 129 | Q754 | Mandibulofacial dysostosis |
| 133 | Q794 | Prune belly syndrome |
| 133 | Q795 | Other congenital malformations of abdominal wall |
| 133 | Q793 | Gastroschisis |
| 133 | Q792 | Exomphalos |
| 134 | Q064 | Hydromyelia |
| 134 | Q068 | Other specified congenital malformations of spinal cord |
| 134 | Q079 | Congenital malformation of nervous system, unspecified |
| 134 | Q049 | Congenital malformation of brain, unspecified |
| 134 | Q069 | Congenital malformation of spinal cord, unspecified |
| 138 | I678 | Other specified cerebrovascular diseases |
| 138 | G934 | Other and unspecified encephalopathy |
| 141 | I424 | Endocardial fibroelastosis |
| 145 | K638 | Other specified diseases of intestine |
| 145 | B332 | Viral carditis |
| 145 | K632 | Fistula of intestine |
| 145 | K519 | Ulcerative colitis, unspecified |
| 145 | B338 | Other specified viral diseases |
| 145 | K928 | Other specified diseases of the digestive system |
| 145 | B341 | Enterovirus infection, unspecified |
| 145 | K566 | Other and unspecified intestinal obstruction |
| 145 | K633 | Ulcer of intestine |
| 145 | K631 | Perforation of intestine (nontraumatic) |
| 145 | B348 | Other viral infections of unspecified site |
| 145 | K565 | Intestinal adhesions [bands] with obstruction (postinfection) |
| 145 | R11 | Nausea and vomiting |
| 146 | P780 | Perinatal intestinal perforation |
| 158 | E770 | Defects in post-translational modification of lysosomal enzymes |
| 158 | E711 | Other disorders of branched-chain amino-acid metabolism |
| 158 | E752 | Other sphingolipidosis |
| 158 | E744 | Disorders of pyruvate metabolism and gluconeogenesis |
| 158 | E778 | Other disorders of glycoprotein metabolism |
| 158 | E740 | Glycogen storage disease |
| 158 | E748 | Other specified disorders of carbohydrate metabolism |
| 158 | E728 | Other specified disorders of amino-acid metabolism |
| 158 | E771 | Defects in glycoprotein degradation |
| 158 | E723 | Disorders of lysine and hydroxylysine metabolism |
| 158 | E720 | Disorders of amino-acid transport |
| 158 | E725 | Disorders of glycine metabolism |
| 158 | E710 | Maple-syrup-urine disease |
| 161 | G931 | Anoxic brain damage, not elsewhere classified |
| 168 | Q614 | Renal dysplasia |
| 168 | Q611 | Polycystic kidney, infantile type |
| 168 | Q613 | Polycystic kidney, unspecified |
| 168 | Q619 | Cystic kidney disease, unspecified |
| 168 | Q615 | Medullary cystic kidney |
| 168 | Q610 | Congenital renal cyst |
| 168 | Q612 | Polycystic kidney, adult type |
| 170 | Q909 | Down syndrome, unspecified |
| 172 | P60 | Disseminated intravascular coagulation of newborn |
| 181 | A415 | Sepsis due to other Gram-negative organisms |
| 181 | A413 | Sepsis due to Hemophilus influenzae |
| 185 | Q230 | Congenital stenosis of aortic valve |
| 190 | P038 | Newborn affected by other specified complications of labor and delivery |
| 199 | Q319 | Congenital malformation of larynx, unspecified |
| 199 | Q313 | Laryngocele |
| 199 | Q321 | Other congenital malformations of trachea |
| 199 | Q315 | Congenital laryngomalacia |
| 199 | Q318 | Other congenital malformations of larynx |
| 199 | Q324 | Other congenital malformations of bronchus |
| 199 | Q322 | Congenital bronchomalacia |
| 199 | Q320 | Congenital tracheomalacia |
| 199 | Q312 | Laryngeal hypoplasia |
| 199 | Q311 | Congenital subglottic stenosis |
| 244 | Q225 | Ebstein's anomaly |
| 281 | P000 | Newborn affected by maternal hypertensive disorder |
